# Utilizing GCN-Based Deep Learning for Road Extraction from Remote Sensing Images

**DOI:** 10.3390/s25133915

**Published:** 2025-06-23

**Authors:** Yu Jiang, Jiasen Zhao, Wei Luo, Bincheng Guo, Zhulin An, Yongjun Xu

**Affiliations:** 1 Institute of Computing Technology, Chinese Academy of Sciences, Beijing 100190, China; jiangyu@ict.ac.cn (Y.J.); u202142776@xs.ustb.edu.cn (B.G.); anzhulin@ict.ac.cn (Z.A.); xyj@ict.ac.cn (Y.X.); 2 University of Chinese Academy of Sciences, Beijing 100049, China; 3 North China Institute of Aerospace Engineering, Langfang 065000, China; luowei@radi.ac.cn

**Keywords:** depthwise separable convolution, graph convolution, road extraction, smart cities, gradient operator, graph reasoning

## Abstract

The technology of road extraction serves as a crucial foundation for urban intelligent renewal and green sustainable development. Its outcomes can optimize transportation network planning, reduce resource waste, and enhance urban resilience. Deep learning-based approaches have demonstrated outstanding performance in road extraction, particularly excelling in complex scenarios. However, extracting roads from remote sensing data remains challenging due to several factors that limit accuracy: (1) Roads often share similar visual features with the background, such as rooftops and parking lots, leading to ambiguous inter-class distinctions; (2) Roads in complex environments, such as those occluded by shadows or trees, are difficult to detect. To address these issues, this paper proposes an improved model based on Graph Convolutional Networks (GCNs), named FR-SGCN (Hierarchical Depth-wise Separable Graph Convolutional Network Incorporating Graph Reasoning and Attention Mechanisms). The model is designed to enhance the precision and robustness of road extraction through intelligent techniques, thereby supporting precise planning of green infrastructure. First, high-dimensional features are extracted using ResNeXt, whose grouped convolution structure balances parameter efficiency and feature representation capability, significantly enhancing the expressiveness of the data. These high-dimensional features are then segmented, and enhanced channel and spatial features are obtained via attention mechanisms, effectively mitigating background interference and intra-class ambiguity. Subsequently, a hybrid adjacency matrix construction method is proposed, based on gradient operators and graph reasoning. This method integrates similarity and gradient information and employs graph convolution to capture the global contextual relationships among features. To validate the effectiveness of FR-SGCN, we conducted comparative experiments using 12 different methods on both a self-built dataset and a public dataset. The proposed model achieved the highest F1 score on both datasets. Visualization results from the experiments demonstrate that the model effectively extracts occluded roads and reduces the risk of redundant construction caused by data errors during urban renewal. This provides reliable technical support for smart cities and sustainable development.

## 1. Introduction

Road information constitutes a critical component in various urban initiatives such as urban renewal, green infrastructure planning, and the development of intelligent transportation systems. It is integral to optimizing resource allocation and enhancing the efficiency of environmentally sustainable urban designs [1,2,3,4]. The task of road extraction (RE), which is approached as a binary semantic segmentation problem, has been acknowledged as both challenging and distinctive. Over the years, numerous methods for RE have been developed, both domestically and internationally. These methods can be broadly categorized into traditional machine learning approaches and contemporary deep learning based techniques. Initially, RE methods predominantly utilized traditional techniques such as threshold-based segmentation and morphological processing. Throughout the first decade of the 21st century, the widespread adoption of machine learning algorithms—including Support Vector Machines (SVMs), Decision Trees, and Random Forests—coupled with the increased availability of computational resources and reduced costs, led to the prominence of machine learning-based methods in RE tasks. These methods leverage remote sensing images and analyze texture, edges, and shape features to perform image segmentation, thus facilitating target extraction. For instance, Ghaziani [5] introduced a method for RE utilizing binary image segmentation, while Unsalan [6] implemented edge detection and a voting mechanism for this purpose. However, traditional RE methods are heavily reliant on manually designed features, which are inadequate for addressing the complexity and dynamic nature of remote sensing data. This limitation restricts their potential applications in the context of smart cities. Additionally, the structural diversity and complexity of road systems, coupled with interference from background elements such as buildings, significantly impede the effectiveness of these traditional machine learning approaches in accurately and efficiently extracting road features.

In recent years, with the rapid advancement of deep learning, the integration of intelligent technologies to achieve precise road extraction and further support urban green renewal and sustainable development has become a shared focus for both academia and industry. Convolutional Neural Networks (CNN), leveraging their powerful feature extraction capabilities, have emerged as a dominant tool for road extraction tasks. Hinton [7] proposed a CNN-based method for road extraction from aerial images. Alshehhi et al. [8] implemented a patch-based CNN model that replaced global average pooling with fully connected layers for simultaneous extraction of roads and buildings from remote sensing imagery. Liu et al. [9] proposed a method utilizing a CNN model to classify aerial imagery and extract road centerlines. Li et al. [10] employed a CNN-based model to extract roads from high-resolution satellite imagery by predicting the probability of each pixel belonging to a road segment, achieving high accuracy in road extraction. Long et al. [11] introduced a Fully Convolutional Network (FCN) that generates prediction maps for pixel-wise segmentation by replacing fully connected layers in CNN with convolutional layers, upsampling the output to the original input size, and fusing it with outputs from intermediate pooling layers. Varia et al. [12] adopted a deep learning technique, specifically the FCN-32 architecture, to extract road segments from very high-resolution UAV (Unmanned Aerial Vehicle) imagery. Despite the impressive efficiency demonstrated by these models in road extraction, they often misclassify non-road areas as roads, particularly in regions of high complexity, leading to a significant number of false negative (FN) errors. Furthermore, FCNs inevitably lose critical edge location information during the process of expanding the receptive field through pooling layers. To better utilize this crucial information, Panboonyuen et al. [13] developed an enhanced deep convolutional encoder–decoder model based on SegNet for segmenting the road category from high-resolution images. SegNet optimizes the encoder–decoder architecture, improving its precision. During the decoding phase, it utilizes the indices saved from the pooling layers in the encoder to recover spatial details at edge locations. While achieving good segmentation results, the lack of contextual information linkage constrained the extraction performance, resulting in poor road connectivity. In subsequent improvements, Mei et al. [14] proposed the Connectivity Attention Network (CoANet) for road extraction from satellite imagery, advancing the technology by capturing long-range dependencies. Zhu et al. [15] designed multi-parallel dilated convolutions and a global context-aware block to predict the contextual spatial relationships of roads, thereby enhancing the structural integrity and connectivity of the extracted results. Wu et al. [16] introduced a node heatmap and bidirectional connectivity-based framework, Bi-HRNet, for satellite image road extraction. This framework addresses the challenge of connectivity prediction difficulties caused by sparse nodes in traditional methods through node heatmaps and bidirectional connectivity classification, while simultaneously improving the topological correctness of node connections. Luo et al. [17] proposed an Auxiliary Decoding Road Extraction Network (AD-RoadNet) that preserves multi-scale road details and enhances connectivity, achieving performance breakthroughs via a dual-module decoupling strategy. Lin et al. [18] presented a road extraction method for complex backgrounds based on multi-scale information fusion and an Asymmetric Generative Adversarial Network (MS-AGAN). Zhang et al. [19] proposed NodeConnect, a method for joint learning of nodes and connectivity for topological road extraction from satellite imagery. By transforming road extraction into a structured prediction problem using graph representation, NodeConnect fundamentally addresses the fragmentation issues inherent in post-processing segmentation results, offering a new paradigm for road network topology extraction. With the widespread integration of attention mechanisms into deep learning models, Transformer-based models have emerged as more effective solutions for the challenge of global context modeling. Liu et al. [20] proposed RoadCT, a satellite image road extraction method based on a CNN–Transformer hybrid network. This hybrid design mitigates the road fragmentation issues associated with pure CNNs and the boundary blurring defects of pure Transformers, achieving a balance between efficiency and accuracy in remote sensing road extraction. Zhu et al. [21] introduced SASwin Transformer, which utilizes spatial self-attention and spatial MLP to optimize feature aggregation.

Despite significant advancements made by these improved models—based on CNN, attention mechanisms, and Transformers—in mitigating road fragmentation, enhancing connectivity, and modeling global context, they remain fundamentally constrained by the indirectness inherent in modeling road topology. CNN-based models primarily process regular grid data. While their convolutional kernels possess powerful feature extraction capabilities within local receptive fields, they struggle to explicitly capture and represent the inherent, non-local topological relationships of road networks. Transformers, while capable of modeling global dependencies, rely on self-attention mechanisms that compute relationships between all pixel pairs. This results in substantial computational overhead and likely involves a large number of redundant interactions unrelated to road topology, making them a less efficient representation for graph structures like roads, which exhibit specific, sparse connectivity patterns. Furthermore, the process of converting pixel-level segmentation results into a coherent topological road network graph itself constitutes an additional, complex post-processing step. This conversion is prone to introducing errors or information loss. Therefore, there is an urgent need for a method that can directly model the inherent graph structure of roads and explicitly learn the spatial dependencies among elements, fundamentally ensuring connectivity and integrity in the presence of occlusions and complex background interference.

Within this technological evolution, Graph Convolutional Networks have demonstrated significant advantages in road extraction tasks due to their unique capability for processing graph-structured data and their enhanced mechanism for focusing on regions with ambiguous features. Road networks inherently possess a graph structure (where nodes represent intersections/road segments, and edges represent connectivity). GCNs can directly model the spatial relationships and topological dependencies between elements, which is crucial for enhancing road connectivity and integrity. The Separable Graph Convolutional Network (SGCN) proposed by Zhou et al. [22] effectively strengthens road feature representation by capturing global contextual information across channels and spatial features. The Dual Attention Network (DANet) by Fu et al. [23] and the Cascaded Attention DenseUNet (CADUNet) by Li et al. [24] demonstrate the potential of combining attention mechanisms with spatial/topological modeling to focus on critical regions and improve road network connectivity. Furthermore, the work by Xu et al. [25] explored the feasibility of combining GCNs with CNNs for feature integration. However, existing GCN-based methods often struggle to restore the continuity of occluded road segments when encountering severe occlusion, leading to fragmented extraction results. Moreover, they are prone to confusion when non-road areas exhibit high similarity to roads in spectral, textural, or even shape characteristics. The key limitation lies in the inadequate utilization of graph structure information. While the adjacency matrix is central to GCNs, current adjacency matrix construction methods fail to effectively integrate underlying semantic similarity information. This restricts the model’s ability to precisely distinguish roads from non-roads in regions with ambiguous features (such as occlusion boundaries or areas near confusing backgrounds) and also constrains the modeling of complex topological relationships.

Therefore, addressing the dual challenges of occlusion interference and background similarity, and inspired by the complementary strengths of Graph Convolutional Networks (GCNs) and Convolutional Neural Networks (CNNs) in efficient feature extraction, along with the advantage of attention mechanisms in feature selection, we propose a novel road extraction method for remote sensing imagery, termed FR-SGCN. This method modifies the graph construction scheme by combining a similarity matrix with the adjacency matrix for graph convolution operations. It employs attention mechanisms to weight the extracted features, accentuating critical information. Furthermore, it introduces a hybrid loss function to mitigate errors caused by class imbalance during model training, thereby effectively enhancing the model’s discriminative capability between roads and visually similar backgrounds. It is noteworthy that while Transformers excel at global modeling, their substantial computational overhead and the characteristic of computing attention across the entire image lead to lower efficiency when processing high-resolution remote sensing imagery. Moreover, they may introduce a significant number of redundant correlations unrelated to local road structures. In contrast, the computational cost of spatial and channel attention mechanisms is significantly lower than the self-attention in Transformers. This makes them far more suitable for embedding within encoder–decoder architectures that need to handle high-dimensional features, particularly when combined with GCNs. Critically, this progressive feature weighting strategy from local to global contexts effectively complements the explicit topological relationship modeling inherent in GCNs. This synergistic combination serves the goals of road connectivity preservation and background differentiation with greater precision.

The contributions of this study are delineated as follows:

1. High-dimensional features are extracted using CNNs, with ResNeXt—characterized by its unique group convolution structure—serving as the primary architecture. This enhances both the model’s accuracy and its feature extraction capabilities while maintaining a constant parameter count.
2. A hybrid loss function is formulated by integrating the stability of binary cross-entropy loss with the optimization prowess of the Dice loss function, effectively addressing the imbalance between background and road features in the images.
3. A feature separation module within the encoder incorporates AMs, facilitating separate extraction of spatial and channel features. Spatial attention is applied during the extraction of spatial features, and channel attention during channel feature extraction, with dynamic weighting of each feature.
4. We have reengineered the adjacency matrix construction within the graph convolution process of the encoder. By merging data from the similarity matrix with the adjacency matrix, constructed using gradient operators, we establish a novel adjacency matrix for graph convolution. This matrix furnishes enriched structural information about the graph, thereby enabling the GCN to consider both structural and feature similarities between nodes concurrently.

The remainder of this study is organized as follows: Section 2 introduces the dataset and the proposed method; Section 3 discusses the experiments, results, and their analysis; Section 4 concludes the study.

## 2. Materials and Methods

### 2.1. Dataset

**Figure 1:**
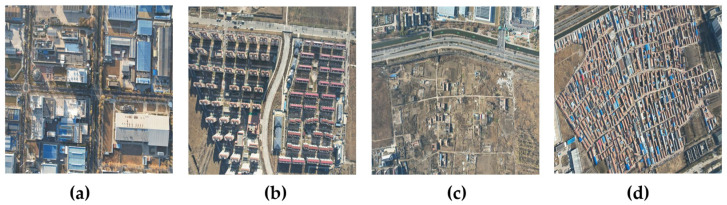
Sample images from the Langfang City dataset, Hebei Province. (**a**) Industrial area, (**b**) Residential area, (**c**) Rural area, (**d**) Densely populated residential area.

Verification experiments employed our self-constructed road dataset of Langfang City, Hebei Province, China, acquired using Unmanned Aerial Vehicle (UAV) sensors (DJI Innovation Technology Co., Ltd., Shenzhen, China); the dataset comprises 11,710 images in total (8192 for training, 1171 for validation, and 2342 for testing), each with dimensions of 1500 × 1500 pixels and a Ground Sampling Distance (GSD) of 0.1 m per pixel. Given the substantial scale and complexity of the dataset, fully manual vectorization of all road masks was impractical. Consequently, a semi-automated workflow leveraging specialized remote sensing software (specifically ArcGIS (Redlands, CA, USA; its business in China is operated by Esri China (Hong Kong) Limited, Beijing, China) Pro’s road tracing tool to generate initial road centerlines) coupled with meticulous human supervision was adopted to efficiently generate high-quality ground truth masks. This involved buffering the extracted centerlines to create initial road polygons, which then underwent extensive manual review and refinement: correcting misalignments between centerlines and visible road centers, adjusting road widths to match local visual appearances, adding missing road segments, removing false detections, ensuring topological correctness, and refining road edges for enhanced precision. A subset of finalized masks underwent secondary review to guarantee dataset consistency and accuracy before rasterization into binary road masks (road pixels = 1, background pixels = 0). Due to the dataset’s suburban location and associated geographic sensitivities, the majority of the data cannot be made publicly available; only a representative subset encompassing Langfang City Industrial Area, Residential Area, Rural Area, and Dense Rural Area is released for reference, with sample images shown in Figure 1.

**Figure 2:**
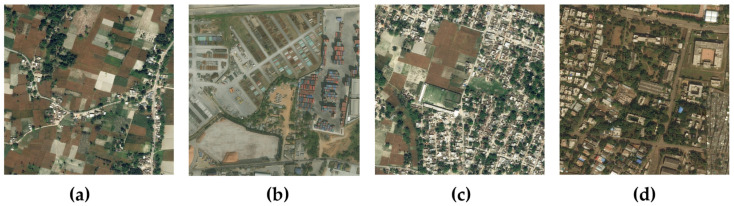
Samples of the DeepGlobe dataset.Both images (**a**,**c**) have obvious tree occlusions, and images (**b**,**d**) have the problem of similar backgrounds.

The DeepGlobe dataset, established by, is an aerial imagery dataset covering regions in Thailand, Indonesia, and India, featuring high resolution and multispectral information that enables the capture of fine surface details. It is primarily used for Earth observation tasks such as object classification and detection, consisting of 6226 images, sized 1024 × 1024 pixels with a spatial resolution of 0.5 m. Since ground truth labels are only available for the training images, we utilized the entire training set as experimental data, randomly partitioning it according to established precedent into 4696 images for training and 1530 images for testing—representing an approximately 75%/25% split—with representative samples of this dataset illustrated in Figure 2.

### 2.2. Structural Overview

**Figure 3:**
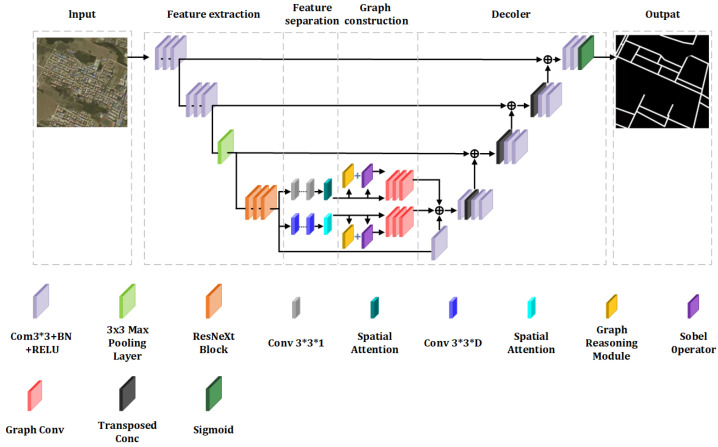
FR-SGCN model architecture.

The RE model presented in this study is structured into four principal components, as illustrated in Figure 3:

1. Feature Extraction: In contrast to the conventional three-channel RGB information, the extraction of high-dimensional features offers a more comprehensive dataset. The ResNeXt architecture is employed to perform this function, capturing an extensive range of data.
2. Feature Separation: Given that spatial and channel features occupy distinct dimensions, their separate extraction facilitates enhanced representational capability. An AM is incorporated to variably weight these features, utilizing split depthwise separable convolutions in conjunction with the AM to precisely delineate both spatial and channel features.
3. Graph Construction: The model incorporates two distinct GCNs to assimilate the global contextual information of the spatial and channel features within the FR-SGCN framework. Initially, a similarity matrix is generated through feature-based graph reasoning, which, when merged with an adjacency matrix derived from gradient operators, forms a new adjacency matrix. Subsequent feature propagation through the GCNs allows for the integration of all features, denoting this sequence as the encoder module.
4. Decoder: Following the GCNs, the model employs transpose convolutions and convolution layers for upsampling, which restores the resolution of the feature map. The fusion of features at various levels is achieved by integrating corresponding features from the encoder module at each stage. The model concludes with convolution layers and a Sigmoid classifier that delineates roads from the background on a pixel-wise basis, effectively categorizing the output into two channels.

#### 2.2.1. Feature Extraction Module

The road extraction network takes RGB images as input; the module processes raw three-channel remote sensing imagery and outputs high-dimensional features for subsequent processing by the feature separation module. This study employs a Convolutional Neural Network (CNN) architecture to acquire these high-dimensional features. Among numerous classic structures, ResNet [26] is the most widely adopted framework in semantic segmentation. Its variant, ResNeXt [27], by employing a structure incorporating grouped convolutions and residual connections, enables learning richer feature representations and possesses enhanced representational capacity. Furthermore, grouped convolutions improve performance by increasing network width without significantly escalating parameter count. This allows ResNeXt to achieve superior performance when handling complex tasks; thus, we selected ResNeXt as the backbone architecture for feature extraction.

Specifically, the input image first passes through two sets of convolutional layers, followed by a 3 × 3 max-pooling layer to downsample the feature maps, reducing their spatial dimensions while preserving essential feature information. Subsequently, we incorporate three ResNeXt blocks. Within each convolutional block, residual connections are utilized to retain information from preceding layers, facilitating the learning of more complex feature representations. These three ResNeXt blocks possess input channel counts of 64, 128, and 256, and output channel counts of 128, 256, and 512, containing 3, 4, and 6 residual blocks, respectively. Following the ResNeXt blocks, the features pass through an adaptive average pooling layer, which transforms the spatial dimensions of the feature maps to (1,1), thereby compressing the spatial information of each feature map into a singular feature representation. Finally, the feature map is flattened into a high-dimensional feature vector for input to the feature separation module.

#### 2.2.2. Feature Separation Module

**Figure 4:**
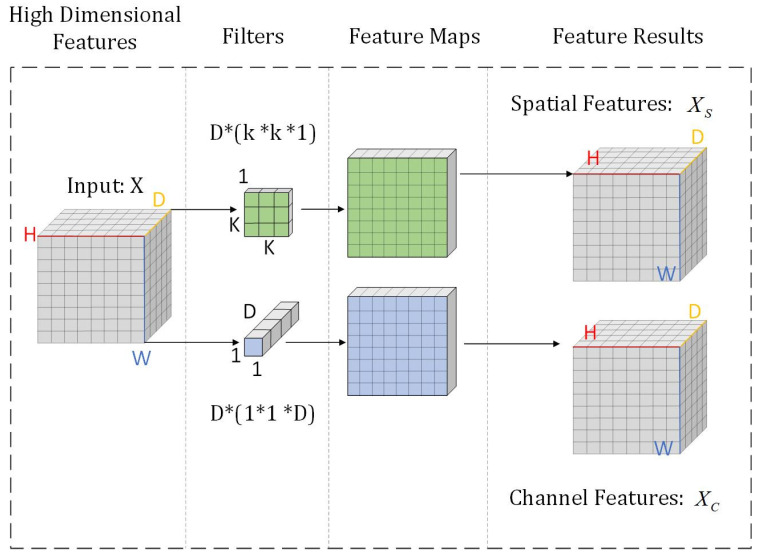
Schematic diagram of the feature classification module.

This module receives the high-dimensional feature maps output by the feature extraction module, aiming to explicitly decouple the features into spatial structural information and spectral channel information to enhance the model’s discriminative capability for road morphology and background. We employ Depthwise Separable Convolution [28] as the fundamental operation due to its effectiveness in reducing computational complexity and parameter count while maintaining feature extraction efficacy. However, distinct from the standard Depthwise Separable Convolution, this study reconfigures the structure. Inspired by the significance of spatial and channel feature distinctiveness for road extraction [29], we explicitly separate the two steps of Depthwise Separable Convolution—Depthwise Convolution (DW Conv) and Pointwise Convolution (PW Conv)—into distinct operations. The DW Conv focuses exclusively on extracting spatial structural information of roads (such as edges, orientation, and connectivity), while the PW Conv concentrates on learning interdependencies between channels and spectral features, as illustrated in Figure 4.

**Figure 5:**
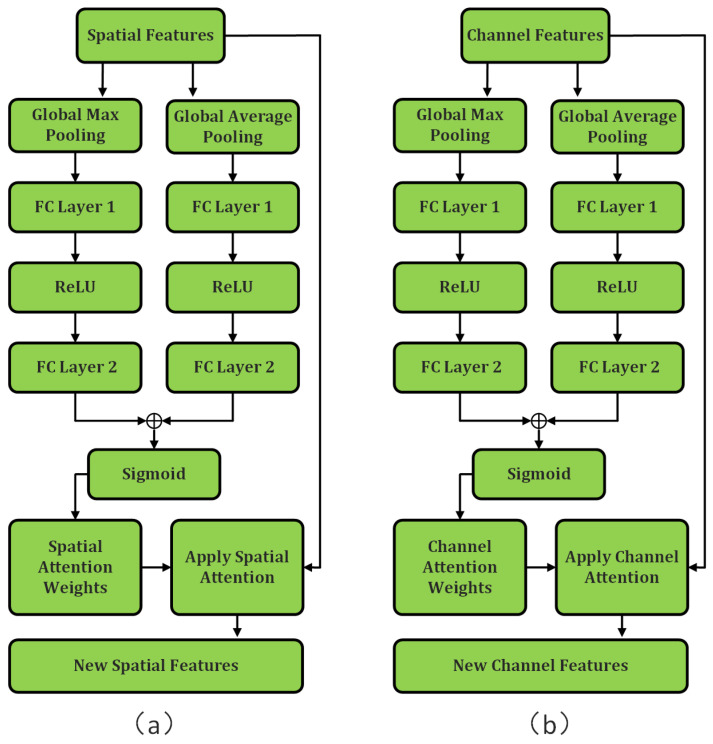
Schematic diagram of the AM: (**a**) Illustration of applying the AM to spatial features post-feature separation. (**b**) Illustration of applying the AM to channel features post-feature separation.

To further enhance the effectiveness of the separated features and focus on critical information, we incorporate attention mechanisms into each path. Following the depthwise convolution path, a spatial attention mechanism [30] is introduced to refine the spatial features. Specifically, two feature descriptors are generated by applying global average pooling and global max pooling to the spatial features $X_{s}$. These descriptors are then processed through two fully connected layers with a ReLU activation function. The resulting outputs are summed and passed through a sigmoid function to obtain the spatial attention weights $A_{s}$. The spatial attention map is applied to the spatial features via element-wise multiplication: ${X_{s}}^{\prime }$ = $A_{s}$\$X_{s}$ (Figure 5), dynamically weighting important spatial locations such as potential road regions and occluded areas. Following the pointwise convolution path, a channel attention mechanism [31] is employed to refine the channel features. Specifically, two feature descriptors are generated by applying global average pooling and global max pooling to the channel features $X_{c}$. These descriptors are processed through two fully connected layers with a ReLU activation function. The resulting outputs are summed and passed through a sigmoid function to yield the channel attention weights $A_{c}$. The channel attention map is applied to the channel features via element-wise multiplication: ${X_{c}}^{\prime }$ = $A_{c}$\$X_{c}$ (Figure 5). This adaptively emphasizes the most discriminative feature channels for road recognition while suppressing background interference channels. These refined feature representations (${X_{s}}^{\prime }$ and ${X_{c}}^{\prime }$) provide enhanced discriminative power for the subsequent graph convolution processing.

#### 2.2.3. Graph Construction

The construction of the graph structure is pivotal for dictating the propagation of information. The framework under discussion innovates by generating a novel adjacency matrix, which integrates a similarity matrix derived from the Road Feature Saliency Graph Reasoning (RFSG) module with another adjacency matrix formulated via gradient operators. This approach employs two distinct Graph Convolutional Networks (GCNs) to enhance the representation of features. The comprehensive architecture is depicted in Figure 3.

The RFSG module encompasses two discrete units that map spatial and road features into defined regions, respectively. Subsequently, the similarity among these regions is quantified, generating the aforementioned similarity matrix [32]. This matrix articulates the similarity relationships among various regions and gauges the saliency of road features. The integration of this similarity matrix with the gradient-based adjacency matrix culminates in a new adjacency matrix. This matrix is instrumental in establishing whether similarity relationships exist between regions based on the saliency levels of the road features. The inclusion of the similarity matrix in the model facilitates guided reasoning across the graph, enabling the effective perception and propagation of information from disparate regions, thereby optimizing the reallocation of road feature weights. This strategy proficiently addresses the challenges associated with the segregation and reconstitution of spatial and channel features. The separated spatial and channel features, outputted from the feature separation module, are inputted into their respective graph reasoning modules as feature maps, denoted as ${X^{\prime }}_{s}$ and ${X^{\prime }}_{c}$, with dimensions H × W × D where H, W, and D represent height, width, and depth, respectively.

Through the implementation of PatchEmbedding, the feature map is subdivided into ($\frac{H}{s}$) × ($\frac{W}{s}$) regions, each measuring *s* × *s* × *C* (where C signifies the number of channels). The features of each region are then vectorized, resulting in a matrix where each row corresponds to the feature vector of a region, with dimensions ($\frac{H}{s}$) × ($\frac{W}{s}$) × (*s* × *s* × *C*). Convolutional processes are applied to extract features, producing a feature matrix $X_{p}\in R^{\left(\frac{H}{s}\right)\times \left(\frac{W}{s}\right)\times \left(s^{2}\times C\right)}$. Each block’s size, $s^{2}\times C$, is subsequently transposed and rearranged to fit the subsequent processing. The block features of each sample are reorganized into matrix form ${\left(X_{p}\right)}^{T}\in R^{\left(\frac{H}{s}\right)\times \left(\frac{W}{s}\right)\times \left(s^{2}\times C\right)}$. Techniques such as global pooling and diagonalization are employed to generate the regional self-connection matrix Λ($X_{p}$).

**Figure 6:**
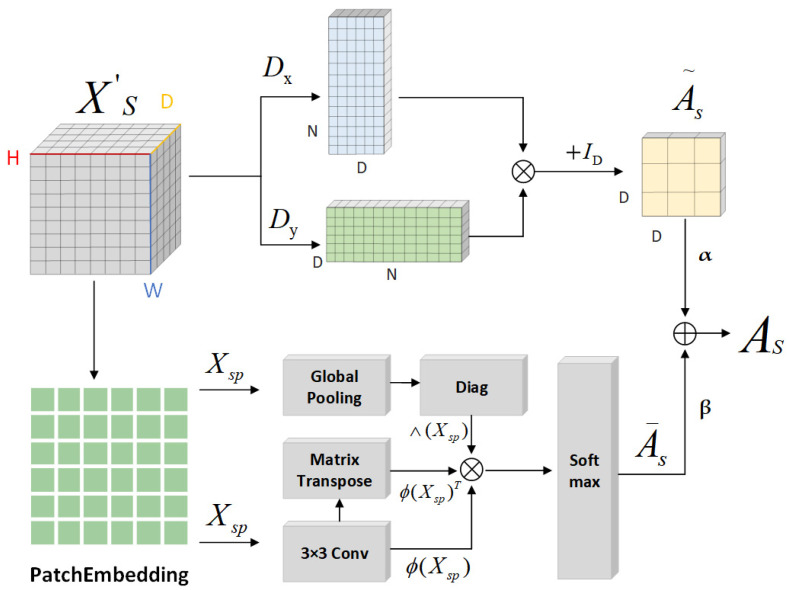
The process of generating a new adjacency matrix for spatial features: the upper section depicts the adjacency matrix creation, while the lower section demonstrates the similarity matrix generation.

**Figure 7:**
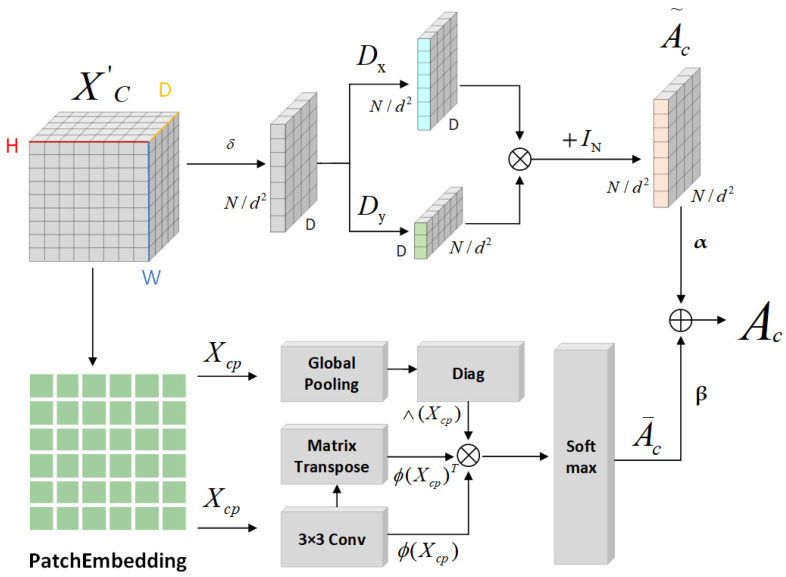
The procedure for forming a new adjacency matrix for channel features: the upper part shows the adjacency matrix production, and the lower part shows the similarity matrix development.

Ultimately, the similarity matrix delineates significant inter-regional relationships. As formulated in Equation (1), and utilizing Softmax for normalization, this matrix calculates the inter-regional feature similarity. Equations (2) and (3) further specify the similarity matrices corresponding to spatial and channel features, respectively. Figure 6 and Figure 7 illustrate the generation of the spatial and channel feature similarity matrices. The matrix also reflects the saliency levels of the road features, particularly highlighting that regions with lower saliency exhibit diminished similarity with other regions.

(1) $$\bar{A}=Softmax\left(\emptyset \left(X_{p}\right)\Lambda \left(X_{P}\right)\emptyset {\left(X_{p}\right)}^{T}\right)$$

(2) $${\bar{A}}_{s}=Softmax\left(\emptyset \left(X_{sp}\right)\Lambda \left(X_{sP}\right)\emptyset {\left(X_{sp}\right)}^{T}\right)$$

(3) $${\bar{A}}_{c}=Softmax\left(\emptyset \left(X_{cp}\right)\Lambda \left(X_{cP}\right)\emptyset {\left(X_{cp}\right)}^{T}\right)$$

${\bar{A}}_{s}$ and ${\bar{A}}_{c}$ are the similarity matrices for the spatial and channel features, respectively. $\emptyset \left(X_{sp}\right)$ and $\emptyset \left(X_{cp}\right)$ denote the spatial feature matrix and channel feature matrix. $\Lambda \left(X_{sP}\right)$ and $\Lambda \left(X_{cP}\right)$ represent the spatial and channel self-adjacency matrices. $\emptyset {\left(X_{sp}\right)}^{T}$ and $\emptyset {\left(X_{cp}\right)}^{T}$ correspond to the transpose matrices of the spatial feature matrix and channel feature matrix (i.e., $X_{sp}$ and $X_{cp}$).

GCNs have advanced the application of convolution operations to graph-structured data by leveraging adjacency matrices. Initially, this approach found utility in the realm of knowledge graphs; however, it has increasingly been applied to extracting features from natural images in recent years. Shi et al. [33] conceptualized each point in *N* (where *N* denotes the number of pixels in the grid coordinates of the input tensor, *N = H × W*) as a vertex, connecting these vertices with edges to adjacent points. Lu et al. [34] employed an adjacency matrix enhanced by a Gaussian kernel function, while Zhang et al. [35] commenced with a randomly initialized adjacency matrix, subsequently optimizing it via gradient descent during the training phase. Liu et al. [36] devised a latent graph through an encoder–decoder architecture. In the context of feature maps $X_{s}$ (spatial features) and $X_{c}$ (channel features), regions exhibiting substantial node value fluctuations typically align with critical structures within the image, encapsulating dense informational content. It is thus rational to link these nodes via edges. For detecting these variations, gradient operators are employed [37]. As illustrated in Figure 6, the spatial adjacency matrix ${\tilde{A}}_{s}$ is constructed based on the input feature map $X_{s}$, featuring dimensions D × D. Correspondingly, the channel adjacency matrix ${\tilde{A}}_{c}$ is derived from the channel feature map $X_{c}$, with dimensions *N × N/*$d^{4}$, where N represents the number of channels and d the downsampling rate. The Sobel operator [38], which includes two kernels, $G_{x}$ and $G_{y}$, facilitates the detection of horizontal and vertical edges, respectively. Application of these kernels to $X_{s}$ and $X_{c}$ produces two gradient maps. The convolution outputs from these maps are then multiplied to construct the adjacency matrix, as depicted in Figure 6 and Figure 7.

(4) $${\tilde{A}}_{s}=G_{x}(X_{s})G_{y}(X_{s})+I_{D}$$

(5) $${\tilde{A}}_{c}=G_{x}(\delta (X_{c}))G_{y}(\delta (X_{c})+I_{N}$$

${\tilde{A}}_{s}$ and ${\tilde{A}}_{c}$ correspond to the spatial adjacency matrix and channel connectivity matrix computed from gradient operators, respectively. $X_{s}$ and $X_{c}$ denote the spatial features and channel features. $G_{x}$ and $G_{y}$ represent the two kernels in the Sobel operator.

In Equation (5), the *δ* operation modifies the positioning of channels within the grid coordinates and executes a downsampling of the channel features, effectively reducing the original channel feature map to a diminished size. Hence, the employment of the Sobel operator not only renders the processing of the channel dimension meaningful but also curtails the parameter count.

The similarity matrix and the adjacency matrix, formulated via the gradient operator, are amalgamated through a weighted addition to generate a novel adjacency matrix. The weight coefficients can be fine-tuned according to the specific application scenario to mirror the significance of various types of connections. Figure 6 and Figure 7 illustrate the comprehensive process of constructing this new adjacency matrix.

(6) $$A_{s}=\alpha {\bar{A}}_{s}+\beta {\tilde{A}}_{s}$$

(7) $$A_{c}=\alpha {\bar{A}}_{c}+\beta {\tilde{A}}_{c}$$

In these equations, $A_{s}$ and $A_{c}$ represent the newly formed adjacency matrices, ${\bar{A}}_{s}$ and ${\bar{A}}_{c}$ denote the similarity matrices, and ${\tilde{A}}_{s}$ and ${\tilde{A}}_{c}$ are the adjacency matrices crafted using the gradient operator, parameterized by the weight coefficients *α* and *β*.

The newly normalized adjacency matrix is incorporated into the graph convolution process to satisfy the computational demands of the GCN.

Graph convolution represents a convolution operation adapted for graph structures, distinguishing it from traditional convolutions that process two-dimensional or three-dimensional tensors. Instead, it involves a graph composed of nodes and edges. By conducting convolution operations between a node and its adjacent nodes, the receptive field is expanded, thereby facilitating the acquisition of contextual information. Following the methodology proposed by Kipf and Welling [39], it is possible to learn deep feature representations of graph-structured data by stacking multiple layers. Within each layer, the features of the nodes are aggregated and updated, enabling the capture of both local and global relationships among the nodes. The graph convolution formula is articulated as follows:

(8) $${\tilde{X}}^{\left(l+1\right)}=\sigma \left({\tilde{D}}^{-\frac{1}{2}}A{\tilde{D}}^{-\frac{1}{2}}X^{l}W\right)$$

where $X^{l}$ represents the node features at the l-th layer, A denotes the normalized new adjacency matrix, $\tilde{D}$ is the degree matrix, *W* is the trainable weight matrix, and *σ* is the nonlinear activation function.

Upon the generation of the new adjacency matrix, a fully connected graph is established, and graph convolution is executed. The feature ${\hat{X}}_{c}$ in Equation (10) is derived by downsampling $X_{c}$.

(9) $${{\tilde{X}}_{s}}^{\left(l+1\right)}=\sigma \left({{\tilde{D}}_{s}}^{-\frac{1}{2}}A_{s}\;\;{{\tilde{D}}_{s}}^{-\frac{1}{2}}{\left({X_{s}}^{T}\right)}^{l}W_{s}\right)$$

(10) $${{\tilde{X}}_{c}}^{\left(l+1\right)}=\sigma \left({{\tilde{D}}_{c}}^{-\frac{1}{2}}A_{c}\;\;{{\tilde{D}}_{c}}^{-\frac{1}{2}}{{\hat{X}}_{c}}^{l}W_{c}\right)$$

where $X^{l}$ indicates the node features at the l-th layer, $A_{s}$ and $A_{c}$ are the new normalized adjacency matrices, ${\tilde{D}}_{s}$ and ${\tilde{D}}_{c}$ are the respective degree matrices, $W_{s}$ and $W_{c}$ are the trainable weight matrices, and *σ* remains the nonlinear activation function.

#### 2.2.4. Decoder

The final refined feature *Y* = ${{\tilde{X}}_{s}}^{\left(3\right)}$ + ${{\tilde{X}}_{c}}^{\left(3\right)}$ + *ψ*(*X*), where ψ undergoes a 3 × 3 convolution before entering the decoder, as depicted in Figure 3. The decoder architecture comprises upsampling, feature fusion, and a final classification layer. Initially, three sets of upsampling operations are performed to progressively enhance the spatial resolution of the feature map to match the size of the original input image. Each upsampling set includes a transposed convolution layer and two convolution layers with 3 × 3 kernel sizes. Following each convolution layer, a batch normalization (BN) layer is applied, which standardizes the features of each batch to expedite training and enhance model stability. The ReLU activation function introduces nonlinearity, which is crucial for learning more complex features. During the feature fusion phase, low-level features, which typically contain more spatial details, are combined with high-level features, which hold richer semantic information, through addition. This integration helps preserve detailed image information and substantially enhances the performance of the segmentation task. The final classification layer consists of a 3 × 3 convolution layer and a Sigmoid classifier. This layer further refines features and prepares them for the final output, with the output channels set to 2, representing road and background. The Sigmoid classifier maps these outputs to a range between 0 and 1, effectively classifying each pixel to distinguish between the road target and background in the final classification.

#### 2.2.5. Hybrid Loss Function

The task of RE requires the classification of pixels within an image into two distinct categories: road and non-road. This classification challenge is essentially a binary classification problem, where the objective is to ascertain whether each pixel is part of the road. The binary cross-entropy loss function [40] is frequently employed to assess the accuracy of the model’s classifications for each pixel. Optimal prediction performance is achieved by minimizing the value of this loss. The binary cross-entropy loss function is defined as follows:

(11) $$L_{BCD}=-\frac{1}{N}\overset{N}{\underset{n=1}{\sum }}\left(y_{n}log\left({y_{n}}^{\prime }\right)+\left(1-y_{n}\right)log\left(1-{y_{n}}^{\prime }\right)\right)$$

Here, $L_{BCD}$ represents the binary cross-entropy loss, *N* is the total number of samples, $y_{n}$ denotes the true label of the n-th sample (where $y_{n}$ = 1 if the pixel is part of the road, and $y_{n}$ = 0 if it is not), and ${y_{n}}^{\prime }$ is the model’s predicted probability that the pixel belongs to the road. This predicted probability should ideally range from 0 to 1.

The extraction of roads from remote sensing images frequently encounters a problem of class imbalance between road pixels and background pixels. The proportion of road pixels relative to the total number of pixels ranges between 5% and 10%. If the model assigns equal weights to road and non-road samples in the loss function, it may exhibit a bias towards the more numerous background pixels, thereby diminishing its ability to detect road pixels and, consequently, reducing the accuracy of RE. In contrast, the Dice loss function [41], which calculates the ratio of the intersection to the union of the predicted results and the true labels, demonstrates superior performance in managing class imbalances. The Dice loss function is defined as follows:

(12) $$L_{DICE}=1-\frac{1}{N}\left(\frac{2\sum_{n=1}^{N}y_{n}{y_{n}}^{\prime }+o}{\sum_{n=1}^{N}y_{n}+\sum_{n=1}^{N}{y_{n}}^{\prime }+o}+\frac{2\sum_{n=1}^{N}(1-y_{n})(1-{y_{n}}^{\prime })+o}{\sum_{n=1}^{N}(1-y_{n})+\sum_{n=1}^{N}(1-{y_{n}}^{\prime })+o}\right)$$

Similarly to $y_{n}$ and ${y_{n}}^{\prime }$, *o* is a constant term.

The Dice loss evaluates the similarity between the expected and predicted outputs for each sample. Its calculation is solely dependent on this similarity and is not influenced by the number of training samples, thus providing a method to balance the proportion of training samples in a specific manner. However, the Dice loss can exhibit abrupt gradient changes during the model training process, potentially leading to unstable training conditions. To mitigate this, a hybrid loss function has been developed, combining the stability of binary cross-entropy loss with the benefits of the Dice loss to circumvent local training optima. The hybrid loss function ($L_{BD}$) is defined as follows:

(13) $$L_{BD}=L_{BCD}+\lambda L_{DICE}$$

In this equation, $L_{BCD}$ is the binary cross-entropy loss, $L_{DICE}$ is as previously described, and *λ* is a coefficient used to balance $L_{BCD}$ and $L_{DICE}$.

## 3. Experimental Results and Analysis

### 3.1. Evaluation Metrics

RE is fundamentally a semantic segmentation challenge. The evaluation of extraction results is conducted using metrics such as recall, precision, and the F1 score. Recall, also referred to as sensitivity, quantifies the model’s capacity to accurately identify positive instances. Within the context of RE, it measures the ratio of correctly identified road areas to the total actual road areas. Consequently, a higher recall indicates that the model captures a greater extent of the road area, thus minimizing the occurrences of missed road regions. Precision evaluates the accuracy of the model in identifying positive instances among those labeled as positive. Specifically, in RE, it measures the ratio of actual road pixels to those predicted as road areas by the model. Thus, higher precision signifies that the predicted road areas are more likely to be true road areas, with fewer false positives involving non-road areas. The F1 score, representing the harmonic mean of precision and recall, serves as a comprehensive metric for assessing the model’s overall performance in identifying road areas. It ranges between 0 and 1, where an F1 score of 1 denotes perfect precision and recall, and a score of 0 indicates extremely poor model performance. A higher F1 score reflects a better overall capability of the model in accurately delineating road areas.

The computation of these metrics is based on the following formulas: recall (Equation (14)), precision (Equation (15)), and the F1 score (Equation (16)).

(14) $$Recall=\frac{TP}{TP+FN}$$

(15) $$Precision=\frac{TP}{TP+FP}$$

(16) $$F1=\frac{2TP}{2TP+FN+FP}$$

In these equations, True Positive (TP) denotes the number of pixels correctly predicted as road areas, False Negative (FN) represents the pixels that are road areas but were not identified as such, and False Positive (FP) signifies the pixels incorrectly labeled as road areas.

### 3.2. Experimental Setup

We conducted our experiments using the PyTorch 1.13.1 framework and accelerated the process with an NVIDIA GeForce GTX 1080 Ti (11 GB) GPU (NVIDIA, Santa Clara, CA, USA). Due to the limitations of GPU memory, each original image was randomly cropped into patches of 512 × 512 pixels. To increase the volume of training data and improve generalization, data augmentation was performed by applying rotations (90°, 180°, and 270°) and flips (horizontal and vertical) to the training samples.

Prior to training, the images were normalized to a range of [−1, 1] employing a method combining 0–1 normalization and mean subtraction, to stabilize gradient computations throughout the training process. All networks utilized the Adam (Adaptive Moment Estimation) optimizer for parameter updates. The Adam optimizer was configured with a learning rate (α) of 10^−3^, decay coefficients (β1 of 0.9 and β2 of 0.999), and a constant (ε) of 10^−8^. The batch size was set at 16.

To evaluate the stability of model results, key experiments (including model comparison and ablation studies on the deep dataset) were repeated five times under identical hyperparameter configurations. Random initialization seeds were set to 1, 2, 3, 4, and 5 using PyTorch’s torch.manual_seed (seed). For each run, we recorded precision, recall, and F1-score metrics. The final reported results represent the mean values with standard deviations across runs. We provide 95% confidence intervals around the means, with variations solely attributable to differences in network parameter initialization. Statistical significance was assessed using two-tailed *t*-tests, with *p*-values reported; *p* < 0.05 indicates statistically significant differences.

### 3.3. Model Comparison Experiments

**Figure 8:**
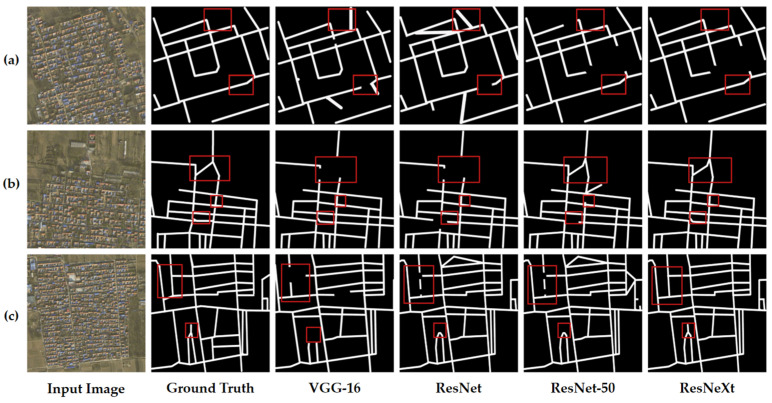
(**a**–**c**) Visualization comparison of different backbone architectures in FR-SGCN. Comparative experiments using different main architectures were conducted on the Langfang City dataset. Red boxes highlight the differences in road extraction results across architectures in complex backgrounds.

In the comparative experiments conducted, we evaluated the efficacy of various backbone architectures for high-dimensional feature extraction, as well as the performance of the proposed model relative to other RE models. Table 1 provides a quantitative comparison of the outcomes of high-dimensional feature extraction leveraging different backbone architectures, and Figure 8 visually represents these results. Similarly, Table 2 offers a quantitative assessment of the RE performance achieved by various methodologies, with corresponding visual results depicted in Figure 8.

For the Langfang city road dataset, the backbone architectures under comparison included VGG-16, ResNet, ResNet-50, and ResNeXt. The results, as detailed in Table 1, indicate that the highest performance in RE was observed with the ResNeXt architecture, achieving a precision of 86.69%, a recall of 78.2%, and an F1 score of 82.22%. These findings substantiate the effectiveness of the backbone network utilized in our methodology.

Figure 8 elucidates the influence of different backbone networks on the RE outcomes for high-dimensional feature extraction, as outlined in Table 1. We selected three representative images, labeled (a), (b), and (c) in Figure 8, for this comparison. These images show scenarios where dense buildings partially obscure roads and where the color and texture of the buildings closely resemble those of the roads. The experimental results reveal that the VGG-16 architecture is prone to gradient vanishing, which leads to a loss of detail and consequently results in incomplete RE in certain areas, as depicted in the red-boxed regions of image (a), where roads are not fully extracted and vegetation is mistakenly classified as road. Similar issues of road segment loss are evident in images (b) and (c). The implementation of residual connections in ResNet mitigates the gradient vanishing issue to some extent. Nevertheless, confusion persists in areas featuring small roads or textures akin to those of roads, highlighted by red boxes in image (a), and instances of road discontinuity are observed in images (b) and (c). Although ResNet-50 enhances RE performance in the red-boxed areas, minor issues persist, such as incomplete extraction of small sections of roads as seen in image (a), and some inaccuracies in road connections in images (b) and (c). ResNeXt significantly outperforms the previous networks in terms of road continuity and accuracy in the red-boxed areas. The RE results in images (a), (b), and (c) within these regions are more closely aligned with the ground truth annotations. This enhancement is attributed to the group convolution and cardinality features of ResNeXt, which facilitate the capture of road features across varying scales and configurations, thereby improving the performance in RE. Based on the outcomes of our comparative experiments, we selected ResNeXt as the optimal backbone architecture for high-dimensional feature extraction in the context of the Langfang city road dataset.

In the Langfang city road dataset, we selected five classical deep learning methods—UNet, SegNet, FCNs, Deeplab V3+, and D-LinkNet34—as well as six state-of-the-art deep learning methods—CoANet, Bi-HRNet, AD-RoadNet, Ms-AGAN, NodeConnect, and RoadCT—for comparison with our proposed method. Table 2 presents a comparative analysis of performance metrics for various RE methodologies applied to the Langfang city road dataset, specifically evaluating precision, recall, and F1-score. Traditional models such as UNet and SegNet exhibit constrained capabilities in feature extraction and face challenges in optimizing the balance between precision and recall. The table illustrates that the UNet model achieves a precision of 82.59%, yet its recall is considerably lower at 63.21%. FCNs demonstrate a marginally inferior performance with an F1 score of 73.01%, although their recall of 68.21% surpasses that of UNet. SegNet records a precision of 77.93%, a recall of 55.25%, and an F1 score of 64.67%. The D-LinkNet34 model registers the highest precision at 88.32% but suffers from a markedly low recall of 45.69%, culminating in an F1 score of merely 60.22%. Deeplab V3+ exhibits the least effective performance, with an F1 score of only 57.01%, accompanied by a precision of 73.53% and a recall of 46.56%. The CoANet method achieved an F1 score of 81.42%, demonstrating robust performance among the state-of-the-art approaches. Its precision (83.89%) and recall (79.10%) are well balanced, indicating an effective trade-off between completeness and accuracy of road extraction. Bi-HRNet exhibited a substantially lower recall (69.50%) compared to the other advanced methods; despite its high precision (83.88%), the low recall yielded an F1 score of only 76.02%. AD-RoadNet performed similarly to CoANet, with an F1 score of 81.18%. It achieved a marginally higher recall (79.11%) but a slightly lower precision (83.37%) than CoANet. Ms-AGAN was the sole model whose recall (78.46%) exceeded its precision (75.66%), resulting in the lowest F1 score (77.03%) among the advanced methods. NodeConnect delivered a strong overall performance, attaining an F1 score of 81.83%, which approaches that of our proposed method; however, its precision (83.34%) remains just below ours. RoadCT achieved a recall of 80.92% and a precision of 83.23%, yielding an F1 score of 82.06%, still inferior to our method—suggesting that RoadCT may sacrifice precision for greater coverage in complex scenes. Our proposed method is the only one to rank first in both key metrics—F1 score (82.23%) and precision (86.69%)—while maintaining a recall (78.20%) within the top three. Compared to the other state-of-the-art models, it exhibits a pronounced advantage in balancing accuracy and completeness.

**Figure 9:**
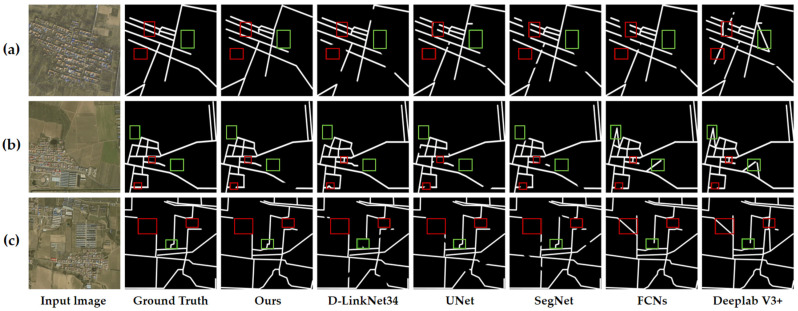
(**a**–**c**) Visual comparison of FR-SGCN and other DL models using the Langfang city road dataset. Performance Comparison Under Partial Occlusion and Low—Contrast Scenarios. Red boxes highlight differences in road extraction results across models under partial occlusion. Green circles demarcate variations in road extraction performance among different models in low-contrast scenarios where backgrounds resemble road surfaces.

**Figure 10:**
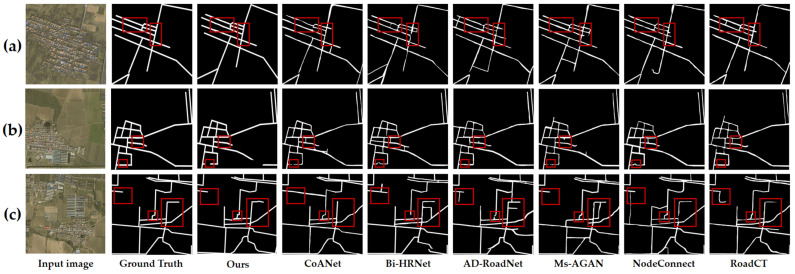
(**a**–**c**) Visual comparison between FR-SGCN and other advanced deep learning models using test data from the Langfang city road dataset. Performance Comparison Under Partial Occlusion and Low—Contrast Scenarios. The red boxes highlight the differences in road extraction results among various models under certain occlusion conditions.

Figure 9 presents a visual comparison of road extraction results obtained by classical models on the test data, while Figure 10 provides a visual comparison of road extraction results produced by advanced models. Three representative images were selected for the comparison. Despite the UNet method not resulting in misclassifications, its skip connections inadequately propagate higher-level semantic information, resulting in missed road regions in the red-boxed areas of images (a) and (b). SegNet demonstrates poor continuity in road predictions, exhibiting numerous discontinuities within the road network across the three experimental images. FCNs manage to extract most road areas but falter in delineating complex roads and handling occlusions. Moreover, the limited receptive field of the fully convolutional structure leads to misclassifications of background noise as roads, as evidenced in the green-boxed regions of image (b). While the dilated convolutions in Deeplab V3+ expand the receptive field, the model struggles to adequately capture local features of narrow roads, and its inefficient decoder design results in the poorest performance, particularly at road edges and fine branches, with misclassifications evident across all three images. The D-LinkNet34 method, despite its high precision, exhibits a low recall with overly conservative predictions that inadequately address small road targets. The red-boxed areas in images (a) and (c) highlight missed detections, particularly where roads are obscured by background interference. The prediction results of our proposed model are the closest to the ground truth, demonstrating high accuracy in road area extraction, particularly in terms of continuity and detail preservation. In contrast, CoANet exhibits local discontinuities in occluded areas, highlighted by red boxes in Figure 10b. The Bi-HRNet method suffers from missed detections, with road segments disappearing within the red boxes in Figure 10a,b. AD-RoadNet performs poorly in low-contrast regions; in Figure 10b, the red box shows excessive edge expansion as the road encroaches into vegetation areas. This is attributed to its adversarial training strategy, which enhances feature representations while weakening spatial constraints. The Ms-AGAN method is affected by shadow interference; due to its generative adversarial network overly focusing on texture similarity, shadows are mistakenly identified as road segments, leading to fragmented false detections, as seen in the red box of Figure 10a. Although NodeConnect achieves good connectivity, it compromises geometric accuracy, resulting in blurred edge details, as observed in the red box of Figure 10c. RoadCT shows adhesion errors, with roads merging into buildings in the red box of Figure 10b. This issue arises from the Transformer’s global attention mechanism, which diminishes local distinctions and confuses roads with rooftops in regions of similar features. In contrast, our method demonstrates superior performance in these areas, producing continuous and complete road extractions without breakage or overextension, and clearly traversing occluded regions. This advantage stems from its graph-based modeling, which integrates similarity and gradient information to effectively reconstruct the topology of occluded roads, as well as from the dual-attention mechanism that dynamically enhances road features while suppressing shadow interference.

Table 3 presents a comparative analysis of road extraction performance between FR-SGCN and six deep learning models—CoANet, Bi-HRNet, AD-RoadNet, Ms-AGAN, NodeConnect, and RoadCT—on the DeepGlobe dataset. The quantitative results demonstrate that FR-SGCN achieves the highest mean F1-score of 82.87%, representing a significant performance improvement over the comparative methods. This is particularly evident when compared with AD-RoadNet (82.18%) and NodeConnect (82.83%), FR-SGCN maintains high precision while exhibiting superior recall capability. In terms of precision, FR-SGCN attains 83.89%, comparable to Bi-HRNet (82.88%) and AD-RoadNet (84.37%), yet with a lower standard deviation (±0.30), indicating enhanced training stability. Regarding recall, FR-SGCN achieves 81.87%, outperforming CoANet (79.10%), Bi-HRNet (77.50%), and AD-RoadNet (80.11%). This superior recall highlights its robustness in extracting roads under complex backgrounds and occlusion scenarios. Although NodeConnect’s F1-score (82.83%) approaches that of FR-SGCN, the *p*-value (0.8828) indicates no statistically significant difference between them. Conversely, all other comparative methods exhibit *p*-values below 0.05, confirming the statistically significant advantage of FR-SGCN over these approaches.

**Figure 11:**
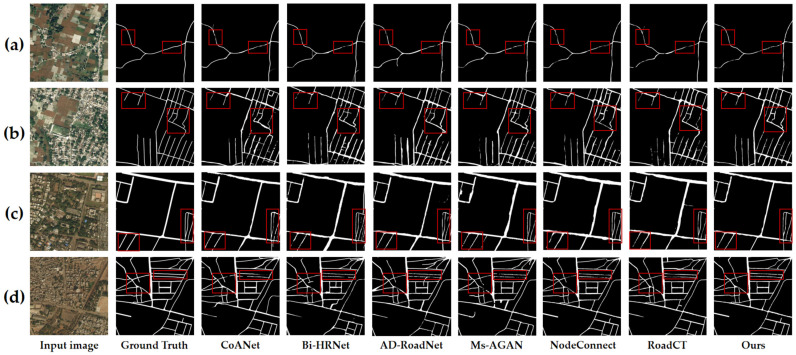
(**a**–**d**) Visual comparison of FR-SGCN and other deep learning models using test data from the DeepGlobe dataset. Performance Comparison Under Partial Occlusion and Low—Contrast Scenarios. Red boxes highlight differences in road extraction results across models under partial occlusion.

Figure 11 presents the visualization results of FR-SGCN compared with six other deep learning models—CoANet, Bi-HRNet, AD-RoadNet, Ms-AGAN, NodeConnect, and RoadCT—on the DeepGlobe dataset. The four selected image patches exhibit challenges including partial road occlusion by tree canopies, low contrast, and interference from similar backgrounds. As shown in the figure, the CoANet and Bi-HRNet methods frequently suffer from disconnections at fine occlusions and intersections, as highlighted by the red boxes in Figure 11a,d. The AD-RoadNet method extracts main roads relatively completely, but tends to exhibit “over-expansion” at the edges of minor branch roads, particularly in the upper-right region of Figure 11d. The Ms-AGAN method produces more noise, with branch roads either misclassified as background or appearing as intermittent pseudo-road segments, thereby compromising connectivity; this issue is marked by the red box in Figure 11c. The NodeConnect method demonstrates superior overall connectivity compared to the preceding methods. The RoadCT method exhibits limited effectiveness in fusing features at intersections. The structure within the red box in Figure 11d appears blurred at road crossings, leading to the loss of detailed path information. In contrast, our proposed method achieves a balance between global context and local details, effectively recovering fine branch roads, intersections, and occluded regions, resulting in optimal connectivity. As visible within the red boxes, our approach avoids both over-expansion and issues like disconnections or missed detections. This performance is attributed to the graph construction module, which generates a novel adjacency matrix by integrating a similarity matrix with gradient operators. This enables the Graph Convolutional Network (GCN) to simultaneously model node relationships based on both feature structure and feature similarity, thereby enhancing its capacity to capture global contextual information. Concurrently, the attention mechanism module dynamically recalibrates the weights of spatial and channel features, allowing the model to focus more effectively on critical features and nodes while mitigating background interference and intra-class variations.

### 3.4. Ablation Experiments

**Figure 12:**
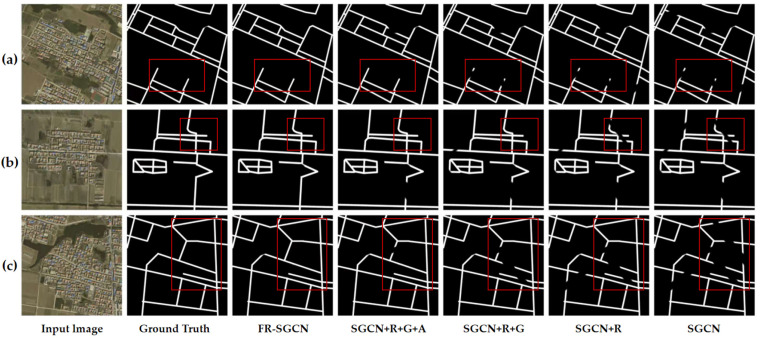
(**a**–**c**) Visual results of the ablation experiments for the proposed method using the Langfang city road dataset. Performance Comparison Under Partial Occlusion and Low—Contrast Scenarios. Red boxes highlight the impact of incorporating individual modules on extraction results in complex backgrounds.

To evaluate the effectiveness of the proposed method, a series of ablation studies were conducted using the newly designed network on the Langfang city road dataset. These experiments primarily assessed three metrics: precision, recall, and F1-score. Consistency in parameter settings and training strategies across all models was maintained to ensure comparability. The outcomes of these studies are documented in Table 4, with corresponding visual results depicted in Figure 12. The results indicate that the proposed method outperforms the baseline network across all metrics.

SGCN + R indicates that the ResNeXt backbone proposed in this work was incorporated into the SGCN baseline model for high-dimensional feature extraction. As shown in Table 4, SGCN + R achieved a precision of 81.55 ± 0.43, a recall of 75.49 ± 0.44, and an F1-score of 78.40 ± 0.41 (mean ± standard deviation over five independent runs). By leveraging ResNeXt for high-dimensional feature extraction, the model’s performance improved, particularly with recall and F1-score increases of 2.08% and 1.41%, respectively. The grouped convolutional structure of ResNeXt strikes an effective balance between parameter efficiency and feature representation capability, thereby significantly enhancing the model’s representational power.

SGCN + R + G indicates that a graph construction module, designed by us, was integrated into the SGCN + R model. As shown in Table 4, the results reveal a precision of 83.29 ± 0.39, recall of 76.97 ± 0.47, and F1-score of 80.01 ± 0.43. These results demonstrate that the addition of the graph construction module further enhanced the model’s performance. Compared to SGCN + R, precision increased by 1.74%, recall by 1.48%, and F1-score by 1.61%. The graph construction module forms a new adjacency matrix by combining a similarity matrix with gradient operators, enabling the GCN to simultaneously consider both structural and feature similarities between nodes. This improvement allows the model to better capture global contextual relationships and enhances its ability to model data structures, resulting in significant improvements in both precision and recall, with a more notable enhancement in precision. This suggests that the graph construction module contributes to more accurate identification of positive samples.

SGCN + R + G + A indicates that an attention module was added to the SGCN + R + G model. As shown in Table 4, the results reveal a precision of 84.37 ± 0.37, recall of 77.82 ± 0.40, and F1-score of 80.96 ± 0.39. These results demonstrate that the addition of the attention module further enhanced the model’s performance. Compared to SGCN + R + G, precision increased by 1.08%, recall by 0.85%, and F1-score by 0.95%. The attention mechanism dynamically weights spatial and channel features, enabling the model to focus more effectively on important features and nodes. This helps alleviate issues such as background interference and class-internal variability in the data, thereby further enhancing the model’s feature representation ability.

SGCN + R + G + A + H indicates that a hybrid loss function was used for training the SGCN + R + G + A model, representing the proposed FR-SGCN method. As shown in Table 4, the results reveal a precision of 86.69 ± 0.32, recall of 78.20 ± 0.34, and F1-score of 82.22 ± 0.33. Since this represents the full method, no *p*-value is provided for comparison against itself. After incorporating the hybrid loss function, the model’s performance significantly improved, particularly in terms of precision and F1-score. Compared to SGCN + R + G + A, precision increased by 2.32%, recall by 0.38%, and F1-score by 1.26%. The hybrid loss function combines the stability of binary cross-entropy loss with the optimization capability of the Dice loss function, allowing the model to better balance different types of errors during training. This effectively addresses the class imbalance issue between background and road information in the images, further enhancing the model’s precision and overall performance.

To further substantiate the efficacy of the proposed method, the results of the ablation experiments were visualized, as illustrated in Figure 12. The visual evidence corroborates the quantitative data presented in Table 4. With the sequential inclusion of modules, the model’s predictive outcomes increasingly converge with the actual ground truth labels. A switch to the ResNeXt backbone resulted in a reduction in the number of detection omissions; however, the continuity of road delineation still requires enhancement (differences are delineated in red boxes in Figure 12). This limitation arises because, despite ResNeXt’s improved feature extraction through grouped convolutions and residual connections, it may not completely capture the intricate structure and contextual nuances of roads, particularly those that are irregularly shaped or obscured. The integration of the graph construction module subsequently adjusted the model’s focus towards road regions, markedly enhancing road continuity. Nonetheless, further refinement is needed in certain details. The incorporation of an AM, which dynamically weights spatial and channel features, compensates for the loss of detail and augments the retention of road details and continuity. Upon implementing the hybrid loss function designed in this study, the ultimate model demonstrates optimal performance in terms of road continuity, detail preservation, and minimization of background distractions, yielding predictions that closely align with the ground truth labels.

The results of the ablation studies affirm that each component within the proposed FR-SGCN method—including the ResNeXt backbone, graph construction module, AM, and hybrid loss function—significantly contributes to the enhancement of the model’s performance. Through the progressive integration of these modules, the model exhibits marked improvements in precision, recall, and F1-score, notably excelling in RE tasks amid complex environments and occlusions. This substantiates the proposed method’s effectiveness and superiority.

Furthermore, ablation studies were performed on various attention mechanisms to validate the effectiveness of our selected approach. Table 5 presents the corresponding ablation results within the FR-SGCN framework. As evidenced in Table 5, our proposed attention mechanism achieves an optimal balance between performance and efficiency. While exhibiting marginally lower precision than Swin Transformer across five independent runs, it attains a competitive mean F1-score of 82.22% ± 0.20-slightly higher than Swin Transformer’s 82.20% ± 0.25, with no statistically significant difference between them (*p* = 0.782). In contrast, the conventional Transformer achieves 81.21% ± 0.30, demonstrating significantly inferior performance to our attention mechanism (*p* < 0.001). This indicates that generic self-attention architectures are less effective for this task than our lightweight design. Moreover, our attention mechanism requires only 12.4 M parameters, representing a 3–4× reduction compared to Transformer (48.7 M) and Swin Transformer (36.5 M). This substantial compression translates to equivalent savings in storage and computational overhead. The efficiency gains stem from its decoupled spatial-channel attention design, which eliminates the quadratic computational overhead inherent in Transformer’s pixel-wise relationship modeling while better accommodating high-dimensional feature processing in remote sensing imagery. Under identical hardware configurations, FR-SGCN integrated with our lightweight attention achieves 32.5 FPS inference speed-74% faster than Swin Transformer (18.7 FPS) and 2.6× quicker than standard Transformer (12.3 FPS). For high-resolution remote sensing applications where real-time processing is critical, our module delivers near-real-time inference capabilities.

**Figure 13:**
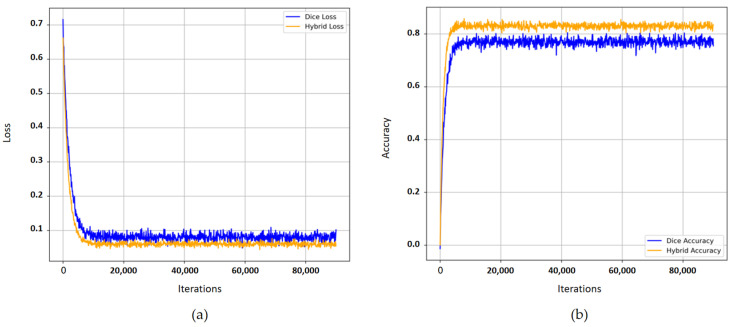
(**a**) Comparison of loss curves and (**b**) comparison of accuracy curves for the two loss functions used during training with FR-SGCN. The two loss functions are the Dice loss and the proposed hybrid loss.

To evaluate the performance improvement of the hybrid loss function on model training, comparative experiments were conducted using both hybrid loss and Dice loss. The results in Table 6 indicate that the hybrid loss function consistently outperforms the standard Dice loss in all evaluated metrics. Over five independent runs, employing Dice loss yields a mean precision of 85.71 ± 0.25%, recall of 77.89 ± 0.30%, and an F1-score of 81.52 ± 0.20%. By contrast, the hybrid loss achieves a mean precision of 86.69 ± 0.20%, recall of 78.20 ± 0.25%, and F1-score of 82.22 ± 0.15%. The difference in F1-score between the two loss functions is highly significant (*p* < 0.001), demonstrating that the hybrid loss not only improves overall accuracy but also reduces variability across runs. Figure 13 displays the loss and accuracy trajectories for both loss functions during the training phase. As depicted in Figure 13a, although the trajectory of the Dice loss function shows a declining trend, it exhibits fluctuations with increasing iterations, indicative of unstable model training. Conversely, the trajectory for the hybrid loss function demonstrates a gradual and stable decline, accompanied by a smoother curve. In Figure 13b, accuracy is initially low but increases swiftly as training progresses; ultimately, the accuracy achieved with the hybrid loss function substantially surpasses that of the Dice loss function, with fewer fluctuations in the later stages. These observations confirm that the hybrid loss function not only enhances the model’s predictive accuracy for RE tasks but also ensures greater stability in model training.

This section presents a series of systematic experiments demonstrating the effectiveness of the FR-SGCN model. On the Langfang dataset, FR-SGCN achieved an F1-score of 82.22%, significantly outperforming benchmark models such as UNet and D-LinkNet34; notably, it maintained road connectivity in regions with occlusions and low-contrast conditions. On the DeepGlobe dataset, FR-SGCN obtained an F1-score of 82.87%. Compared to deep-learning methods such as CoANet, RoadCT, and AD-RoadNet, the *p*-values were all below 0.05, indicating that the performance gains are statistically significant. Visualizations further confirm the model’s robustness to canopy occlusion and connectivity breaks at road intersections. The ablation studies, conducted by incrementally adding each module, clearly quantify the contribution of each component. Furthermore, we conducted additional ablation experiments on the attention mechanism and loss function. The lightweight attention mechanism, with only 12.4 M parameters, achieves an F1-score comparable to that of the Swin Transformer (36.5 M parameters) while reaching an inference speed of 32.5 FPS. In addition, the hybrid loss function improves the F1-score by 0.7% compared to the Dice Loss and produces more stable training curves. These results indicate that FR-SGCN, through the collaborative optimization of multiple modules, effectively addresses the challenges inherent in remote-sensing road extraction.

## 4. Conclusions

This study addresses the challenging problem in remote-sensing road extraction wherein background regions bearing visual similarity to road surfaces degrade extraction accuracy. We propose an improved graph convolutional network (GCN) model, termed FR-SGCN, which adopts the following design. First, high-dimensional features are extracted via a ResNeXt backbone and subsequently fed into a feature-separation module before entering a graph-construction module. This encoder architecture more effectively captures global contextual information. Within the feature-separation module, both spatial-attention and channel-attention submodules are integrated, yielding more distilled spatial and channel representations. In the novel graph-construction module, we introduce a technique that fuses feature-similarity and gradient information to build adjacency matrices. This enables roads with weak visual cues to receive equal attention, resulting in more accurate and continuous extraction under occlusion and complex environmental conditions. Additionally, a hybrid loss function is designed to mitigate class-imbalance issues, thereby improving training stability. Qualitative results demonstrate that FR-SGCN offers significant advantages for remote-sensing road-image extraction. Quantitatively, on our road dataset’s test split, FR-SGCN achieves 86.69% precision, 78.20% recall, and an 82.22% F1-score—surpassing all comparative models and yielding the best overall performance. On the DeepGlobe benchmark, it attains 83.89% precision, 81.87% recall, and an 82.87% F1-score. Although its precision is marginally lower than that of AD-RoadNet and NodeConnect, FR-SGCN produces the most balanced performance, achieving the highest F1-score of 82.87%. Visualization results confirm that FR-SGCN effectively overcomes challenges posed by background–road similarity and discontinuities in extracted road networks. Finally, we discuss and validate the effectiveness of the proposed method. Beyond technical innovation in model design, this work offers a scalable intelligence solution for urban renewal and green, sustainable development, significantly enhancing road detection capabilities in areas of complex terrain and dense vegetation to support eco-friendly infrastructure. Future work will explore further model lightweighting and introduce more robust dynamic-inference mechanisms to bolster generalization in diverse urban environments, thereby advancing the integration of intelligent technologies with sustainable development.

## Figures and Tables

**Table 1:** Final classification results of feature extraction using different backbone architectures on the Langfang city road dataset.

Backbone	Precision	Recall	F1-Score
VGG-16	84.88%	70.2%	76.85%
ResNet	85.26%	75.2%	79.91%
ResNet-50	85.78%	77.3%	81.94%
ResNeXt	**86.69%**	**78.2%**	**82.22%**

**Table 2:** Road extraction results using different methods on the Langfang city road dataset.

Method	Precision	Recall	F1-Score
UNet	82.59%	63.21%	71.61%
SegNet	77.93%	55.25%	64.66%
FCNs	78.51%	68.21%	73.00%
Deeplab V3+	73.53%	46.56%	57.02%
D-LinkNet34	**88.32%**	45.69%	60.22%
CoANet	83.89%	79.10%	81.42%
Bi-HRNet	83.88%	69.50%	76.02%
AD-RoadNet	83.37%	79.11%	81.18%
Ms-AGAN	75.66%	78.46%	77.03%
NodeConnect	83.34%	80.38%	81.83%
RoadCT	83.23%	80.92%	82.06%
Ours	86.69%	**78.20%**	**82.23%**

**Table 3:** Road extraction results of different methods on the DeepGlobe road dataset. * Mean F1-scores and standard deviations are computed over five independent runs, with 95% confidence intervals reported. *p*-values indicate statistical significance derived from comparisons against our method’s F1-score. Asterisk (*) denotes *p* < 0.05, signifying statistically significant differences relative to FR-SGCN.

Method	Precision (Mean ± Std)	Recall (Mean ± Std)	F1-Score (Mean ± Std)	*p*-Value
CoANet	82.89% ± 0.45%	79.10% ± 0.50%	80.95% ± 0.47%	0.00012 *
Bi-HRNet	82.88% ± 0.40%	77.50% ± 0.60%	80.10% ± 0.50%	0.00002 *
AD-RoadNet	**84.37% ± 0.35%**	80.11% ± 0.55%	82.18% ± 0.45%	0.02660 *
Ms-AGAN	82.66% ± 0.50%	79.46% ± 0.48%	81.03% ± 0.49%	0.00021 *
NodeConnect	84.34% ± 0.42%	81.38% ± 0.52%	82.83% ± 0.47%	0.88280
RoadCT	83.23% ± 0.38%	80.32% ± 0.50%	81.75% ± 0.44%	0.00240 *
Ours	83.89% ± 0.30%	**81.87% ± 0.40%**	**82.87% ± 0.35%**	

**Table 4:** Ablation study results of the FR-SGCN method. * Mean F1-scores and standard deviations are computed over five independent runs, with 95% confidence intervals reported. *p*-values indicate statistical significance derived from comparisons against the F1-score of the full FR-SGCN method. Asterisk (*) denotes *p* < 0.05, indicating statistically significant differences relative to the complete FR-SGCN framework.

Method	Precision (Mean ± Std)	Recall (Mean ± Std)	F1-Score (Mean ± Std)	*p*-Value
SGCN	80.94% ± 0.40%	73.41% ± 0.45%	76.99% ± 0.38%	0.00012 *
SGCN + R	81.55% ± 0.43%	75.49% ± 0.44%	78.40% ± 0.41%	0.00002 *
SGCN + R + G	83.29% ± 0.39%	76.97% ± 0.47%	80.01% ± 0.43%	0.02831 *
SGCN + R + G + A	84.37% ± 0.37%	77.82% ± 0.40%	80.96% ± 0.39%	0.00240 *
SGCN + R + G + A + H (FR-SGCN)	**86.69% ± 0.32%**	**78.20% ± 0.34%**	**82.22% ± 0.33%**	

**Table 5:** Ablation study results of different attention mechanisms in the FR-SGCN method. * Mean F1-scores and standard deviations are computed over five independent runs, with 95% confidence intervals reported. *p*-values indicate statistical significance derived from comparisons against the F1-score of our attention mechanism. Asterisk (*) denotes *p* < 0.05, indicating statistically significant differences relative to our attention mechanism.

Method	Precision (Mean ± Std)	Recall (Mean ± Std)	F1-Score (Mean ± Std)	*p*-Value	Parameters (M)	Inference Speed (FPS)
Transformer	85.52% ± 0.35%	77.15% ± 0.40%	81.21% ± 0.30%	0.00036 *	48.7	12.3
Swin Transformer	**86.72% ± 0.30%**	77.83% ± 0.35%	82.20% ± 0.25%	0.78201	36.5	18.7
Ours	86.69% ± 0.25%	**78.20% ± 0.30%**	**82.22% ± 0.20%**		**12.4**	**32.5**

**Table 6:** Ablation study results of different loss functions in the FR-SGCN method. * Mean F1-scores and standard deviations are computed over five independent runs, with 95% confidence intervals reported. *p*-values indicate statistical significance derived from comparisons against the F1-score of our hybrid loss function. Asterisk (*) denotes *p* < 0.05, indicating statistically significant differences relative to our hybrid loss function.

Method	Precision (Mean ± Std)	Recall (Mean ± Std)	F1-Score (Mean ± Std)	*p*-Value
Dice Loss	85.71% ± 0.25%	77.89% ± 0.30%	81.52% ± 0.20%	0.00025 *
Hybrid Loss	**86.69% ± 0.20%**	**78.20% ± 0.25%**	**82.22% ± 0.15%**	

## Data Availability

Due to privacy concerns associated with the filming area of this study, the entire dataset cannot be made publicly available. Nevertheless, a subset of this dataset is shared via Baidu Netdisk (https://pan.baidu.com/s/1keSzPghXpM9tUbw6ACJ9eA?pwd=ri73 accessed on 30 April 2025) without privacy risks.
